# The Effects of Food Labelling on Postexercise Energy Intake in Sedentary Women

**DOI:** 10.1155/2017/1048973

**Published:** 2017-05-25

**Authors:** Jacynthe Lafrenière, Jessica McNeil, Véronique Provencher, Éric Doucet

**Affiliations:** ^1^Behavioural and Metabolic Research Unit, School of Human Kinetics, University of Ottawa, Ottawa, ON, Canada; ^2^Institute of Nutrition and Functional Foods, Laval University, Quebec City, QC, Canada

## Abstract

Food labelling has been previously reported to influence energy intake (EI). Whether food labels influence postexercise EI remains to be determined. We assessed how food labelling and exercise (Ex) interact to influence food perception and postexercise EI. In this randomized crossover design, 14 inactive women participated in 4 experimental conditions: Ex (300 kcal at 70% of VO_2peak_) and lunch labelled as low in fat (LF), Ex and lunch labelled as high in fat (HF), Rest and LF, and Rest and HF. The lunch was composed of a plate of pasta, yogurt, and oatmeal cookies, which had the same nutritional composition across the 4 experimental conditions. EI at lunch and for the 48-hour period covering the testing day and the following day was assessed. Furthermore, perceived healthiness of the meal and appetite ratings were evaluated. There were no effects of exercise and food labelling on EI. However, meals labelled as LF were perceived as heathier, and this label was associated with higher prospective food consumption. Initial beliefs about food items had a stronger effect on healthiness perception than the different food labels and explain the positive correlation with the amount of food consumed (*ρ* = 0.34, *P* < 0.001).

## 1. Introduction

To maintain stable body weight and composition in response to increases in energy expenditure (EE) from exercise, an equivalent increase in caloric intake and/or a decrease in the energy cost of daily activities must also occur [1]. This is defined as complete (100%) postexercise energy compensation. Numerous studies have evaluated the effects of exercise on energy intake (EI), but results are conflicting with studies reporting increases in EI following exercise [2–4], while others did not [5, 6].

The perception of food items is based on socially promoted preconceptions of their impacts on health and weight, and this can exert influence over food choices and ultimately on EI [7, 8]. Foods presented as low in fat or rich in nutrients often carry the reputation of being healthier regardless of the portion size presented [9, 10]. As presented by Chandon and Wansink [11], this cognitive bias is represented by a categorical classification of food items, which has been coined the “health halo” and can lead to overconsumption of foods perceived as healthy. Along these lines, Provencher et al. [12] showed that individuals consumed more cookies when they were described as a healthy snack as opposed to when they were presented as a gourmet treat. Initial knowledge and perception of food items could thus potentially alter projected feelings of fullness and, therefore, the amount of ingested food and consequently calories [13].

In parallel, it has also been reported that individuals overestimate the energy cost of exercise while underestimating the caloric content of ad libitum food intake [14]. According to Spence et al. [15], exercise could also be associated with “guiltless eating,” a concept that refers to the prevalent belief that EE from exercise can offset the EI of a larger meal. As such, in addition to the labelling of food, the perception of EE derived from exercise could also impact postexercise EI, at least acutely.

Discrepant results in postexercise EI between studies could be partly explained by differences in the usual level of physical activity. High physical activity participation tends to be related to a relatively stable weight over time, meaning that EI is closely matched to EE from exercise in these individuals [16]. Moreover, active individuals seem to be more sensitive and responsive to appetite signals, which allow them to maintain energy balance throughout the day [17, 18]. Of interest to the study of postexercise EI is the observation that inactive women could be more prone to increasing food intake following a bout of exercise [4] and are also seemingly more responsive to food labelling [19, 20]. As such, investigating the effects of food labelling on postexercise EI in sedentary women is warranted.

The aim of this study was thus twofold. First, we wanted to determine whether food labelling interacts with exercise to influence EI, the perceived healthiness, and appetite both acutely and for a 48-hour period in sedentary women. Secondly, we aimed to assess whether variations in EI correlated with variations in health perception of the meals. First, we hypothesized that food presented as low in fat (LF) would (1) be consumed in larger amounts independently of exercise and (2) would be perceived as healthier. Second, it was hypothesized that an increase in EI would only be observed following the exercise session from the meal labelled as high in fat (HF) as a result of “guiltless eating” that contributes to partly explaining postexercise energy compensation. Third, it was hypothesized that a healthier perception rating would be associated with an increase in EI.

## 2. Experimental Methods

### 2.1. Participants

Twenty women aged between 18 and 40 years were recruited through ads posted on the University of Ottawa campus. In order to be included, they had to be premenopausal, free from metabolic disorders, and inactive (<150 minutes of moderate to vigorous physical activity/week, which is below the recommendations put forth by the Canadian Society for Exercise Physiology (CSEP)) [21]. Physical activity level was self-reported by participants. Irregular menstrual cycle was also an exclusion criterion given that all testing sessions were performed during the follicular phase of the menstrual cycle. This was done to minimize the impact of hormonal variations on appetite-related variables, since appetite, food intake, and frequency of food craving have been shown to fluctuate across the menstrual cycle [22, 23]. Moreover, participants were not included if they had variations in body weight of more than ±2 kg over the last 6 months. Candidates who smoked, consumed greater than 2 alcoholic drinks per day, took medication affecting food intake (e.g., corticosteroid), had food allergies, and were enrolled in a weight loss diet at the time of the study were also excluded from the study. All selected participants provided written informed consent. Of note is the fact that the true objectives of the study were only divulged when participants had completed all experimental sessions. None of the participants had identified the true objectives of the study. At the onset of the protocol, they were told that the objective was to evaluate variations in appetite and post-workout recovery according to the fat content of the meal. From the 20 participants who met the inclusion criteria, 14 completed all experimental sessions. Their results are presented hereafter. All procedures were approved by the University of Ottawa's ethics committee and are in accordance with the Declaration of Helsinki.

### 2.2. Procedures

#### 2.2.1. Overall Experimental Design

Participants were first invited to a preliminary session during which descriptive data were collected. This study used a crossover design with the four experimental conditions, which were randomly assigned in a counterbalance order (Rest + Meal labelled LF; Rest + Meal labelled HF; Exercise + Meal labelled LF; and Exercise + Meal labelled HF).

#### 2.2.2. Preliminary Session

During the preliminary session, the consent form was reviewed and signed by participants. At this time, anthropometric measurements were taken and maximal aerobic capacity was determined, as the intensity of exercise during the experimental session was set at 70% of each participant's maximal oxygen consumption (VO_2peak_), which was determined using the VO_2_ reserve method [24]. This is considered to be high intensity exercise [25] and was associated with postexercise energy compensation in women [3]. For VO_2peak_, a progressive stress test to exhaustion was completed under the supervision of an exercise physiologist using the Canadian Society for Exercise Physiology protocol [26]. The grade on the treadmill was increased progressively every two-minute until exhaustion, whereas the speed was constant for the duration of the test. A Vmax 229 series metabolic cart (SensorMedics Corporation, Yorba Linda, CA) was used to collect breath-by-breath samples of expired air through a mouthpiece, from which VO_2_ was obtained. Heart rate (Polar RS300X, Kempele, Finland), as well as perceived exertion assessed with the Borg scale [27, 28], was closely monitored throughout the test. Participants were then also asked to fill out the Three-Factor Eating Questionnaire (TFEQ) [29] to determine the level of dietary restraint, disinhibition, and hunger.

#### 2.2.3. Experimental Sessions

Participants were asked to arrive at the laboratory following a 12-hour overnight fast and to avoid alcohol consumption, as well as intense physical activity participation (e.g., training, playing sports) for at least 24 hours prior to the start of each experimental session. These instructions were sent by e-mail before each session and compliance was confirmed on the morning of all 4 experimental sessions. For the first session only, participants were instructed to arrive at the laboratory at 7:00 a.m. following a 12-hour overnight fast for a measurement of resting metabolic rate (RMR) (Vmax Encore 29N metabolic cart (SensorMedics Corporation, Yorba Linda, CA)). Data were collected for a 20-minute period after 20 minutes of rest in the supine position and after a 5-minute acclimatization period under the ventilated hood. During this first experimental session, participants chose the foods they wanted for breakfast from a list of items on a validated food menu [30]. The generic name of the different food item was shown, with no other nutritional information. Each food item consumed during the first session was weighed and then used as a standardized breakfast for the next three experimental sessions. For the remaining 3 experimental sessions, participants were asked to arrive at 8:00 a.m. They were served exactly the same breakfast as that they had eaten during the first experimental session and had to consume everything. Although the amount and type of foods varied from one participant to the next, it is important to stress that each participant received exactly the same breakfast for all experimental sessions.

From 8:30 a.m. to 10:45 a.m., participants were allowed to do any sedentary activities of their choice in the laboratory. During 2 of the 4 experimental sessions, an exercise session (Ex) consisting of walking with an incline or running on a treadmill was performed (preceded by a proper warm-up of 5 minutes at 3.5 mph). The intensity was set at 70% of each participant's VO_2peak_, as determined during the preliminary session. During this session, heart rate was monitored to determine the intensity of the Ex based on the linear relationship that exists between VO_2_ and heart rate under the anaerobic threshold [31, 32]. Participants exercised until they had expended an estimated 300 kcal. The required duration of the Ex was calculated using the VO_2_ that corresponded to 70% of the VO_2peak_ obtained during the preliminary session. The Weir equation was used to calculate the caloric equivalent of this VO_2_ measure [33, 34] (mean duration of the Ex was 33 ± 5.4 minutes). During the resting sessions (Rest), participants were asked to stay seated in the laboratory and had to remain seated for the same duration as the Ex intervention (i.e., 33 ± 5.4 minutes). At 11:45 a.m., for all conditions, participants were required to take a 15-minute shower and were then offered the experimental lunch. Before leaving the laboratory, at 12:30, participants chose what they would like to eat for the remainder of the day as well as for the day that followed the experimental manipulations.

### 2.3. Anthropometric Measurements

Height was measured using a Tanita HR-200 height rod (Tanita Corporation of America, Inc., Arlington Heights, IL) and weight was assessed with a standard beam scale (HR-100, BWB-800AS, Tanita Corporation, Arlington Heights, IL). Body composition was determined using dual-X-ray absorptiometry (DXA) scanner (Lunar Prodigy, General Electric, Madison, WI). Body composition was only measured at baseline 2 hours into the postprandial period, whereas weight was assessed at the beginning of each experimental session, following a 12-hour overnight fast.

### 2.4. Energy Intake Assessment

Lunch was served at noon and included pasta with cream sauce, vanilla flavoured yogurt, and oatmeal cookies (see Table 1). The same meal was offered at each of the four experimental sessions and all three-food items were provided in ad libitum fashion. Because all women were tested during the follicular phase of the menstrual cycle, at least 1 month separated all experimental conditions. The characteristics of the meal were presented on a piece of paper and read to participants by the experimenter. On two occasions (Rest and Ex), the foods were described as “high in fat (HF)” using the following description: (1) pasta: pasta with creamy vegetable sauce made with 35% cream and a rich cheese, (2) yogurt: vanilla flavoured yogurt made with creamy whole milk, and (3) cookies: gourmet oatmeal cookies made with real butter and old-fashioned brown sugar. And on the two other occasions (Rest and Ex), this same meal was provided but presented as “low in fat (LF)” with the following labels: (1) pasta: whole-wheat pasta with fresh vegetables in a light sauce made with reduced fat cream and cheese, (2) yogurt: nonfat vanilla flavoured yogurt, and (3) cookies: cookies made with fibre rich whole-grain oatmeal. Participants were instructed to eat as much or as little as they wanted of each of these items over a 30-minute period. After lunch, participants received a standardized menu containing 61 food items [30] and were asked to select what they wanted to eat for the remainder of the day as well as for the following day. We opted to measure food intake over 48 hours because it has been shown that exercise and labelling have an influence on EI over a period lasting more than one meal [3, 35]. Participants were instructed not to eat food outside of those selected from the menu for the measurement period (the remainder of the day and the following day). The selected food items were prepared according to guidelines previously described by McNeil et al. [30]: that is, all foods were weighed and stored in coolers to allow participants to carry them home. They were also instructed to bring back all leftovers and packaging two days later. EI for the entire measurement period is available for 12 participants, as we were unable to obtain EI during the last 24 hours in two participants who moved to a new city just after the last experimental session and were unable to bring back the coolers. It is important to note that information for the meal that followed Ex was nonetheless available for these two participants.

### 2.5. Appetite and Food Perception Measurements

Before and after each meal, as well as before the Ex or the Rest sessions, participants were asked to complete 100 mm visual analogue scales (VAS) to assess their desire to eat, hunger, fullness, and prospective food consumption [36, 37]. Additionally, during lunch, participants were asked to rate their appreciation of each item presented during the lunch meal on how much they liked it using a visual analogue scale (anchored from “not at all” to “very much”). They were also asked to provide a score from 1 to 8 on the following questions: “how healthy does this plate look to you?” (1 = “very unhealthy”; 8 = “very healthy”), “If you were eating this plate regularly, how would it affect your weight?” (1 = “weight loss”; 8 = “weight gain”), and “Do you consider this plate as appropriate for a healthy menu?” (1 = “very inappropriate”; 8 = “very appropriate”). These questions were adapted from those used by Provencher et al. [12].

### 2.6. Statistical Analysis

Statistical analyses were performed using SPSS software (version 17; SPSS Inc., Chicago, IL). Normality of the distribution was first assessed using the Kolmogorov-Smirnov test. A two-way repeated measures analysis of variance (ANOVA) was used to determine the main effect of exercise (Ex/Rest) and meal labelling (HF/LF) on EI at lunch, as well as for the entire measurement period (standardized breakfast, experimental lunch, and ad libitum out-lab intake assessed with the standardized menu for the remainder of the day and the following day). The effect of exercise (Ex/Rest) and labelling (HF/LF) on the perceived healthiness (i.e., “healthiness,” “effect on weight,” and “appropriateness in a healthy menu” ratings) for each food item (pasta/yogurt/cookies) was assessed with a nonparametric Friedman's test as the scale used is an ordinal variable and the distribution was not normal. To adjust for multiple tests performed, we employed a Bonferroni correction and as a result the level of significance was set at *P* ≤ 0.005. When nonparametric Friedman's test was found to be significant, Wilcoxon tests were used to find where significant differences existed. Paired *T*-tests were carried out to explore if Ex affected appetite (i.e., desire to eat, hunger, fullness, and prospective food consumption) before lunch. Two-way repeated measures ANOVA with exercise (Ex/Rest) and labelling (HF/LF) as within subject factors were used to assess the effects and interactions of these factors on appetite after the lunch. Finally, in order to describe variation in EI, Spearman's rank order correlations (*ρ*) were carried out to assess the relationships between perception ratings at lunch.

## 3. Results

### 3.1. Participant Characteristics

Participant characteristics are shown in Table 2. Body weight remained stable across experimental sessions (*P* = 0.738).

### 3.2. Energy Intake

The average EI for the standardized breakfast was 609.8 SD 163.6 kcal (24.8% of the energy intake of the day), and as mentioned in the methods each participant consumed the same breakfast at every experimental session. Energy and macronutrient intakes at lunch across sessions are shown in Table 3. There was no main effect of Ex (*P* = 0.541) or food labelling (*P* = 0.984) on EI at lunch. Moreover, contrary to our hypothesis, no significant interactions were noted between Ex and food labelling (*P* = 0.248). The effect size of this outcome was small (partial *η*^2^ = 0.101; observed power: 20%) [38]. Fat-free mass, fat mass, and RMR were included in the model as covariate because they are known to be important predictors of EI [39], but they did not demonstrate a significant cofounding effect and neither did participants' restraint, disinhibition, and hunger levels as assessed by the TFEQ. EI (*F*(3,39) = 0.203 *P* = 0.894) and appreciation of the foods (degree to which participants liked the meal on a scale from “not at all” to “very much”) were not significantly affected by the order of experimental sessions. Energy and macronutrient intakes over 48 hours were not different across sessions (Table 3).

### 3.3. Appetite Rating

There was no effect of Ex on prelunch appetite ratings. However, a significant main effect of Ex (*F*(1,7) = 7.252 *P* = 0.018) and of food labelling (*F*(1,7) = 4.697 *P* = 0.049) on prospective food consumption after lunch was observed. More specifically, participants reported being able to eat more food after Ex as well as after the LF meal sessions (Rest + LF: 8.7 SD 8.2 mm; Rest + HF: 6.3 SD 6.6 mm; Exercise + LF: 13.1 SD 8.4 mm; Exercise + HF: 10.4 SD 7.8 mm on a 100 mm scale), although they did not significantly eat more during the rest of the day.

### 3.4. Perceived Healthiness

There was no effect of Ex on healthiness rating (*P* = 0.240), on the perceived effects on weight (*P* = 0.360), or on the appropriateness in a healthy menu (*P* = 0.173). Food items labelled as LF were globally perceived as healthier (*P* < 0.001) and more appropriate in a healthy menu (*P* < 0.001) than food items labelled as HF. However, labels did not influence perceived effect on weight (*P* = 0.180).  Table 4 presents perceived healthiness ratings for each item across experimental conditions. The Friedman test revealed that labelling did not affect perceived healthiness of cookies the same way as it did on the other food items. Indeed, no significant difference was observed in the perceived healthiness rating (*χ*^2^(3) = 11.713 *P* = 0.006) and the capacity to induce weight gain (*χ*^2^(3) = 10.705 *P* = 0.01) across conditions (using the 0.005 level of significance adjusted for multiple comparisons) suggesting that the perception of the cookies was not affected by label manipulations. The yogurt was globally perceived as healthier (*P* < 0.001), more appropriate in a healthy menu (*P* < 0.001), and less associated with weight gain (*P* < 0.001) when compared with pasta and cookies.

### 3.5. Association between Perceived Healthiness and Food Consumption

Correlation analyses demonstrated that a higher rating of perceived healthiness was associated with a reduced perceived capacity to affect weight (*ρ* = −0.747, *P* < 0.001) and higher ratings of the appropriateness in a healthy menu (*ρ* = 0.831, *P* < 0.001). A positive correlation was also observed between the perceived healthiness rating and the weight of food consumed (g) when all three-food items were pooled together (*ρ* = 0.339, *P* < 0.001), though no significant correlation was found between the perceived healthiness and EI (kcal). The appreciation rating was also positively associated with an increased consumption of pasta (*ρ* = 0.472, *P* < 0.001) and cookies (*ρ* = 0.267, *P* = 0.047).

## 4. Discussion

We set to investigate whether food labelling interacts with exercise to influence postexercise EI, the perceived healthiness of meals, and appetite in sedentary women. We also assessed whether variations in EI correlated with variations in health perception of the meals. To our knowledge, this is the first study to evaluate the effects of both exercise and food labelling on postexercise EI within the same study design. Contrary to our hypothesis, neither exercise nor labelling the lunch as HF or LF had an impact on EI acutely (lunch) or for the 48-hour testing period. However, as hypothesized, food items labelled as LF were perceived as healthier and associated with higher prospective food consumption after the meal. We also show that the food items with the highest score for perceived healthiness were consumed in larger amounts as shown by the positive correlation between perceived healthiness rating and the weight (g) of the different food item consumed.

A number of studies have shown that a bout of exercise does not acutely impact EI [6, 40], which is in accordance with the results presented in the current study. Indeed, no significant differences were observed for EI at lunch, after Ex or Rest, or for the total 48 hours, meaning that the participants did not compensate for the EE induced by the Ex. Ex only slightly influence appetite perception after lunch. Indeed, participants reported being able to eat a larger amount of food after Ex than after Rest, even if they did not.

Unlike Shide et al., Wooley [41], and Provencher et al. [12] who observed an increase in EI after the ingestion of a food labelled as LF or healthier, no differences in EI were observed between the different fat content labelling in the present study. In accordance with our results, Bowen et al. [42] did not observe any effects of labelling (HF versus LF) on milkshake consumption in a group of women, which is similar to the results reported by Gravel et al. [43] in a mixed population. The methodological diversity of the different study protocols complicates the comparison of study results as far as the impact of labelling on EI is concerned. It is important to mention that, contrary to most of the protocols currently published on the influence of labelling on food intake [8, 12], a complete meal was manipulated and used in our study to assess EI at lunch, compared to single item like milkshakes, yogurt, or cookies. Our results thus illustrated that even if label does influence healthiness perception of food, initial knowledge about nutritional characteristics of the items would exceed the impact of label. Also, those characteristics seem to be further highlighted when different items are presented side by side. As reported by Oakes and colleagues [7, 44], food items often carry the reputation of being good or bad for health and body weight control, and this preconception strongly influences perception and caloric estimations of the product. The lunch meal proposed to the participants in our protocol contained food items with good (yogurt), neutral (pasta), and bad (cookies) reputations to ensure a better representation of real life food variety. Food items were also different in terms of their energy density (0.8 kcal/g for the yogurt versus 4.58 kcal/g for the cookies; see Table 1). This distinction explains why we observed a significant correlation between the weight of food consumed (g) (but not EI (kcal)) and perceived healthiness. We found that the labelling affected the perceived healthiness of a product, but this rating varied substantially between the different food items, which reiterated the importance of testing complete meals and not just focusing on single food items.

As hypothesized, the items labelled LF were perceived as healthier, but this manipulation had more impact on the perception of the pasta and the yogurt than on that of the cookies, which were perceived as unhealthy no matter how they were labelled. Buckland et al. [45] demonstrated that a diet-congruent starter (e.g., a salad) was associated with a significant reduction in EI at lunch when compared with a hedonic starter (e.g., garlic bread) in dieters. The labelling manipulation thus likely had reduced impact on the cookies because they are not a diet-congruent food item. It is also in accordance with the conclusion of recent review from Provencher and Jacob [46] which states that labelling influences health perception but that the impact on energy intake is less consistent.

*Limitations*. Although the crossover protocol used for this project is robust and experimental conditions were rigorous and well controlled, limitations need to be mentioned. First, the sample size is relatively small and limited to young women, mostly university students, which limits the generalizability of the results. A posteriori power analysis revealed that a sample of 78 individuals would have been needed to obtain a power of 0.80 [47] and statistically different effects on the average difference between session, which were between 10 and 60 kcal pour EI at lunch. Although the study was indeed underpowered to detect a difference in EI of this magnitude, the clinical relevance of such a difference must also be put into perspective. There is always a possibility that the appreciation of the meal decreased across sessions, as the same meal was presented each time, but as the sessions were randomized with at least one month separating each session, the likelihood of this occurring is substantially reduced. In fact, no effect of the order of the sessions was noted on EI and appreciation at lunch as presented in the results. We also must stress the acute nature of the present study design. Caution is warranted before extrapolating short-term outcomes to long-term exposure, particularly when dealing with energy balance outcomes.

## 5. Conclusion

In summary, labelling a meal as HF or LF did not alter postexercise EI. However, we show for the first time that Ex did influence the effect of labelling on perception, even if actual EI was not significantly changed. Future study designs should assess the effects of the presentation of a wider range of food items on their perception and intake. It would also be important to assess whether EI is affected when manipulations of labelling are maintained over longer periods of time, where it may lead to variations in energy balance and, ultimately, energy stores.

## Figures and Tables

**Table 1:** Energy and macronutrient composition of the experimental lunch.

	Energy (kcal/100 g)	Macronutrients (g/100 g)		
		Carbohydrate	Proteins	Lipids
Pasta plate	95.3	13.1	2.9	2.8
Vanilla yogurt	80.0	12.0	4.9	1.3
Oatmeal cookies	458.3	70.8	4.2	18.8

**Table 2:** Participants characteristics at baseline (*N* = 14).

	Mean (±SD)	Range
Age (years)	22.4 ± 2.7	19–28
Weight (kg)	68.9 ± 12.4	55.6–94.4
BMI (kg/m^2^)	24.1 ± 3.6	18.2–31.9
Fat mass (kg)	22.2 ± 9.2	11.7–45.2
Fat-free mass (kg)	43.0 ± 4.4	34.6–51.2
Body fat (%)	33.0 ± 7.4	20.3–48.3
RMR (kcal/d)	1310.1 ± 124.3	1147.05–1585.9
VO_2peak_ (ml/kg·min)	40.8 ± 6.1	30.3–51.3
TFEQ score (/51)	22.1 ± 8.0	9–34

BMI, body mass index; RMR, resting metabolic rate; and TFEQ, Three-Factor Eating Questionnaire.

**Table 3:** Lunch and total (48 hours) energy (kcal) and macronutrient (g) intake for all four experimental conditions.

	RL	RH	EL	EH	*P* ^c^		
					Exercise status	Labelling	Exercise status^*∗*^ labelling
*Lunch energy intake *(kcal)^a^	622.5 ± 186.1	660.0 ± 186.1	685.0 ± 190.2	648.3 ± 201.5	0.541	0.984	0.248
Carbohydrate (g)^a^	93.1 ± 28.7	94.9 ± 27.2	98.5 ± 28.0	93.1 ± 29,5	0.766	0.567	0.445
Lipids (g)^a^	19.1 ± 6.2	19.5 ± 5.9	20.4 ± 6.7	19.5 ± 6.2	0.676	0.744	0.544
Proteins (g)^a^	20.0 ± 6.4	21.0 ± 6.0	21.3 ± 4.7	19.7 ± 6.4	0.975	0.714	0.129
*Total energy intake *(kcal)^b^	4276.2 ± 1193.5	4215.1 ± 665.1	4377.9 ± 823.7	4671.4 ± 993.3	0.182	0.605	0.301
Carbohydrate (g)^b^	592.2 ± 204.3	551.5 ± 86.5	574.4 ± 121.6	631.2 ± 152.9	0.299	0.792	0.129
Lipids (g)^b^	155.5 ± 56.9	155.2 ± 54.7	167.2 ± 43.1	173.8 ± 38.6	0.188	0.692	0.541
Proteins (g)^b^	145.2 ± 45.1	143.7 ± 27.5	152.9 ± 42.4	157.3 ± 40.3	0.190	0.846	0.619

Means ± s.d.

^a^ *N* = 14; ^b^*N* = 12. *∗* indicates the interaction.

^c^ *P* value for the main effects and interaction of the ANOVA.

Sessions: RL: Rest and low fat; RH: resting and high fat; EL: Exercise and low fat; EH: Exercise and high fat.

Total energy intake includes the self-selected standardized breakfast, the experiment lunch, and energy intake outside the laboratory (total of 48 hours).

**Table 4:** Perceived healthiness rating of the lunch for all four experimental conditions (*N* = 14).

	RL	RH	EL	EH	Exact sig. (*P*)			
					Between condition differences	Labelling	Exercise status	Pairs differences
*Healthiness (/8(SD))*								

Pasta	5.9 (1.2)	3.8 (1.4)	5.5 (1.3)	4.2 (1.5)	<0.001^*∗*^	<0.001^*∗*^	0.488	RL-RH RL-EH RH-EL EL-EH

Yogurt	6.4 (1.0)	4.8 (1.4)	6.4 (1.7)	4.7 (1.8)	0.001^*∗*^	<0.001^*∗*^	0.5	RL-RH RL-EH

Cookies	3.9 (1.5)	3.3 (1.9)	3.6 (1.3)	2.6 (1.3)	0.006	NA	NA	NA

*Effect on weight (/8(SD))*								

Pasta	4.7 (1.1)	6.4 (1.2)	5.0 (0.7)	6.1 (1.1)	<0.001^*∗*^	<0.001^*∗*^	0.478	RL-RH RL-EH RH-EL EL-EH

Yogurt	4.1 (0.9)	5.1 (1.1)	3.9 (0.9)	5.1 (1.2)	<0.001^*∗*^	<0.001^*∗*^	0.247	RL-RH RL-EH RH-EL EL-EH

Cookies	5.8 (1.3)	6.5 (1.3)	5.9 (1.2)	6.6 (1.1)	0.01	NA	NA	NA

*Appropriateness in a healthy menu (/8(SD))*								

Pasta	6.1 (1.1)	3.5 (1.7)	5.4 (1.3)	3.7 (1.8)	<0.001^*∗*^	<0.001^*∗*^	0.169	RL-RH RL-EH RH-EL EL-EH

Yogurt	6.7 (1.2)	4.9 (1.7)	6.5 (1.6)	4.6 (1.7)	0.003^*∗*^	<0.001^*∗*^	0.223	RL-RH RL-EH

Cookies	3.9 (1.6)	2.6 (1.2)	3.9 (1.6)	3.0 (2.0)	0.002^*∗*^	0.001^*∗*^	0.477	RL-RH

Between condition differences are tested with Friedman test.

Main effect and pairs differences are tested with Wilcoxon test only when Friedman test is found to be significant. ^*∗*^*P* < 0.001.

Bonferroni correction is used to correct for multiple analysis and as a result the level of significance is set at *P* ≤ 0.005.

## Abbreviations

ANOVA: Analysis of variance
BMI: Body mass index
CSEP: Canadian Society of Exercise Physiology
RH: Resting and high fat session
RL: Resting and low fat session
Rest: Resting session
DXA: Dual X-ray absorptiometry
EE: Energy expenditure
e.g.: Example
EI: Energy intake
EH: Exercise and high fat session
EL: Exercise and low fat session
Ex: Exercise session
HF: High fat label
i.e.: In other words
Kcal: Kilocalorie
Kg: Kilogram
Min: Minute
Mph: Miles per hour
LF: Low fat label
*P*: Probability that observed data are consistent with the null hypothesis
RMR: Resting metabolic rate
s.d.: Standard deviation
VAS: Visual analogue scales
VO_2peak_: Measured peak oxygen consumption.
